# Mating precedes selective immune priming which is maintained throughout bumblebee queen diapause

**DOI:** 10.1186/s12864-019-6314-9

**Published:** 2019-12-10

**Authors:** Thomas J. Colgan, Sive Finlay, Mark J. F. Brown, James C. Carolan

**Affiliations:** 1 School of Biological, Earth and Environmental Sciences, University College Cork, Cork, County Cork Ireland; 2 School of Biological and Chemical Sciences, Queen Mary University of London, Mile End Road, London, E1 4NS UK; 3 Department of Zoology, School of Natural Sciences, Trinity College Dublin, Dublin 2, Ireland; 4 Centre for Ecology, Evolution and Behaviour, Department of Biological Sciences, Royal Holloway University of London, Egham, Surrey, TW20 0EX UK; 5 Department of Biology, Maynooth University, Maynooth, County Kildare, Ireland

**Keywords:** Diapause, Immunity, Mating, Mass spectrometry-based proteomics, Bumblebees, Pollinator health

## Abstract

**Background:**

Understanding the mechanisms by which organisms adapt to unfavourable conditions is a fundamental question in ecology and evolutionary biology. One such mechanism is diapause, a period of dormancy typically found in nematodes, fish, crustaceans and insects. This state is a key life-history event characterised by arrested development, suppressed metabolism and increased stress tolerance and allows an organism to avoid prolonged periods of harsh and inhospitable environmental conditions. For some species, diapause is preceded by mating which can have a profound effect on female behaviour, physiology and key biological processes, including immunity. However, our understanding of how mating impacts long-term immunity and whether these effects persist throughout diapause is currently limited. To address this, we explored molecular changes in the haemolymph of the ecologically important pollinator, the buff-tailed bumblebee *Bombus terrestris*. *B. terrestris* queens mate prior to entering diapause, a non-feeding period of arrested development that can last 6–9 months. Using mass-spectrometry-based proteomics, we quantified changes in the pre-diapause queen haemolymph after mating, as well as the subsequent protein expression of mated queens during and post-diapause.

**Results:**

Our analysis identified distinct proteome profiles associated with diapause preparation, maintenance and termination. More specifically, mating pre-diapause was followed by an increase in the abundance of antimicrobial peptides, key effectors of the immune system. Furthermore, we identified the elevated abundance of these proteins to be maintained throughout diapause. This finding was in contrast to the general reduction observed in immune proteins during diapause suggestive of selective immune priming and expression during diapause. Diapause also affected the expression of proteins involved in cuticular maintenance, olfaction, as well as proteins of unknown function, which may have roles in diapause regulation.

**Conclusions:**

Our results provide clear molecular evidence for the consequences and benefits of mating at the immune level as it precedes the selective increased abundance of antimicrobial peptides that are sustained throughout diapause. In addition, our results provide novel insights into the molecular mechanisms by which bumblebees prepare for, survive, and recover from diapause, insights that may have implications for our general understanding of these processes in other insect groups.

## Background

The preparation for, as well as maintenance of life within harsh climates, places enormous selective pressures on organisms, resulting in the evolution of adaptations to survive environmental extremes [1, 2]. One such adaptation is diapause, a dormancy-like state whereby organisms reduce or suppress metabolism and can arrest other important biological processes, such as development or reproduction [3–5]. The capacity for diapause is widespread across evolutionarily diverse taxa and has been suggested to contribute to their ability to exploit seasonal resources, as well as bridge periods of unfavourable environments (e.g. winter) [6]. For insects, within any given species diapause is restricted to one of several life stages, including different larval instars and pupal stages, as well as the adult stage of the life-cycle [6, 7]. Most species of diapausing insects can enter diapause in the absence of environmental cues but, more commonly, changes in environmental variables, such as photoperiod or temperature, lead to the preparation and programming of diapause [4, 8–10].

Regardless of the developmental stage of diapause entry, the dormancy-like state consists of three ecophysiological phases including pre-diapause (preparation and induction), diapause (initiation, maintenance and termination), and post-diapause, where development is reinitiated [8]. The expression of the diapause phenotype is regulated by ecdysteroids, maternal effects, and altered gene expression [3, 4] resulting in arrested development, suppressed metabolism, increased longevity and enhanced stress tolerance [3, 5, 11]. However, despite our increasing knowledge of certain physiological processes during diapause, such as reproduction and metabolism, our understanding of other key processes, such as immunity, remains limited [12, 13]. The reduced mobility and metabolic activity of diapausing individuals can place them at increased risk of pathogen infection, which may affect their survival rates and/or affect post-diapause fitness. This is coupled with constitutive immune expression being costly to generate and maintain [14–17], especially during periods of low nutritional intake [18, 19]. Diapausing organisms are also at greater risk of desiccation and require mechanisms to reduce water loss [20–22].

An additional consideration is the effect of mating on the preparation and maintenance of diapause, which in general can have profound long-term effects on female physiology affecting behaviour, immunity and longevity. In relation to diapause, both sexes of some insects undergo diapause with copulation occurring after termination of diapause, in others only females undergo diapause with mating occurring before diapause [23]. During mating, males transfer sperm into the female reproductive tract, where it is stored in a specialised organ for use post-diapause [24–26]. Males may also transfer additional compounds during copulation that modulate aspects of female physiology such as receptivity to remate [27, 28] or immune expression [29–33]. While mating can suppress aspects of the female immune response, in species where mating occurs before diapause this may incur a survival cost, and thus sexual selection might be expected to act on males to ensure that mating does not decrease, or perhaps even increases the likelihood of the female surviving diapause, to ensure paternity. However, at present, our understanding of the long-term effects of mating during diapause is limited.

An exemplary model to explore the prolonged effect of mating on immune expression during diapause is the bumblebee. Bumblebees are key ecological and commercial pollinators providing ecosystem services that maintain biodiversity [34] and improve agricultural crop yields [35]. Like other social Hymenoptera, caste differentiation is present [36], whereby the majority of females, known as workers, perform altruistic tasks, such as resource foraging, brood care, nest maintenance and defence [37], while generally a single reproductive individual, the queen, establishes the colony before transitioning primarily to egg-laying [38]. Despite morphological similarities between queens and workers in certain species, queens often display different behavioural and physiological repertoires in comparison to workers [36] and are the only colony members to undergo diapause [38]. Before diapause, virgin queens, known as gynes, emerge from the natal colony to find a mate. For most bumblebee species, queens mate with just one male [39, 40] before entering diapause, a non-feeding period that can last 6–9 months [41, 42]. Mating in the buff-tailed bumblebee, *Bombus terrestris*, results in an increased transcript expression of antimicrobial peptides, which coincides with increased resistance of mated queens to the pathogenic trypanosome, *Crithidia bombi* [43] demonstrating the immune benefit of mating. However, it is unknown whether this heightened immunity is maintained throughout bumblebee diapause and indeed, after, where queens are also susceptible to infection [44]. Previous studies have identified the role of the endocrine juvenile hormone (JH) [45] and genome-wide changes in transcriptional regulation associated with key metabolic processes, as well as nutrient storage and stress response [46], in the maintenance of bumblebee diapause. While transcriptomics provides valuable insights into gene expression, mRNA or transcript quantification is often a weak to modest proxy for the estimation of the abundance of proteins, which at the biochemical level perform functions directly related to the survival and fitness of an organism, especially during periods of stress [47].

Techniques, such as mass spectrometry-based proteomics, simultaneously identify and quantify thousands of proteins in a biological sample, which enables the assessment of environmental and/or physiological changes at the molecular phenotype level. In addition, RNA approaches are not conducive to the direct assessment of the protein constituents of biological fluids such as haemolymph. In this study, we characterised the haemolymph, a nutrient-rich plasma-like medium present in the haemocoel (open circulatory tract) of arthropods, which is responsible for the transport and delivery of nutrients to tissues [48, 49]. The haemolymph also contains proteins with roles in immunity [50–53], development [54–56], olfaction [48, 57] and neurotransmission [58, 59]. Profiling changes in haemolymph protein abundance has the potential to resolve global molecular signatures and biomarkers underlying key life stage events [59–61], including diapause [62]. Here, using mass spectrometry-based proteomics, we aim to establish a clear understanding of the molecular mechanisms underlying the ecophysiological stages of onset, maintenance and termination of diapause, by profiling the haemolymph proteome throughout diapause for the buff-tailed bumblebee, *B. terrestris*.

In our experiment, we specifically address three key questions. Firstly, how does the queen haemolymph proteome change as a result of mating, and are these changes maintained throughout and after diapause? Secondly, given that diapause represents a period of presumed metabolic inactivity, can changes in protein abundance, particularly of those associated with immune/defence systems and metabolic processes, provide insight into how the diapausing organism prepares for, survives and recovers from diapause. Finally, can the regulatory components of diapause be discerned and identified from queen haemolymph and do they concur with those identified in recent studies using transcriptomic analyses [46, 63] highlighting conserved genetic regulatory mechanisms across insect taxa. To address these questions, we quantified changes in the haemolymph proteome composition at six stages of the queen life-cycle, including two pre-diapause stages (virgin gynes and mated pre-diapause queens), two diapause stages (1 week and 14 weeks during diapause) and two post-diapause time-points (6 h and 48 h post-diapause). Our study reveals novel changes pre-, during and post-diapause in the queen haemolymph proteome highlighting the effect of mating on the queen molecular phenotype, as well as providing key insights into the molecular mechanisms underlying the regulation and expression of the diapause phenotype itself.

## Results

### Haemolymph profiling reveals altered diapause phenotype

In total 129 unique multiple-peptide supported haemolymph proteins were identified from the virgin and mated queens collected across the six time-points examined (Additional file 1: Table S1) of which 79 were identified to be statistically significant differentially abundant (SSDA) following multivariate analysis (ANOVA, FDR < 0.05) (Additional file 2: Table S2a).

**Fig. 1:**
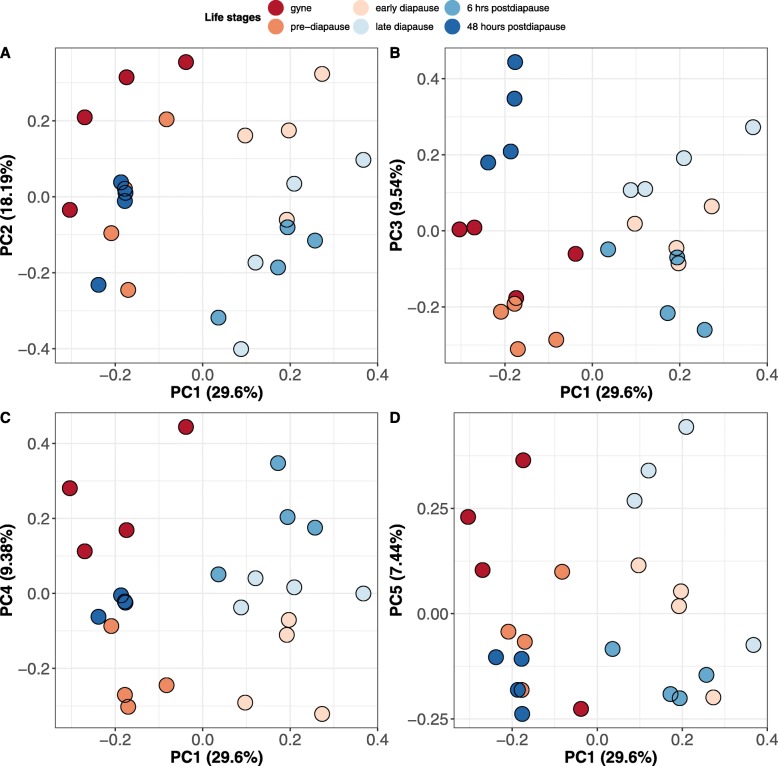
Label-free quantitative proteomic analysis of bumblebee queen haemolymph at six points within the life-cycle. Principal component analysis (PCA) of Z-score normalised label-free quantification (LFQ) intensity values for all statistically significant differentially abundant (SSDA) proteins (*n* = 79) identified (**a**) the first two components (PC1 and PC2) to explain 29.6 and 18.19% of the variance within the dataset, respectively. To further resolve differences in the haemolymph proteome, additional principal components (**b**) PC3, (**c**) PC4, and (**d**) PC5 were plotted individually against the first principal component. Each queen life stage is indicated by a unique colour

**Fig. 2:**
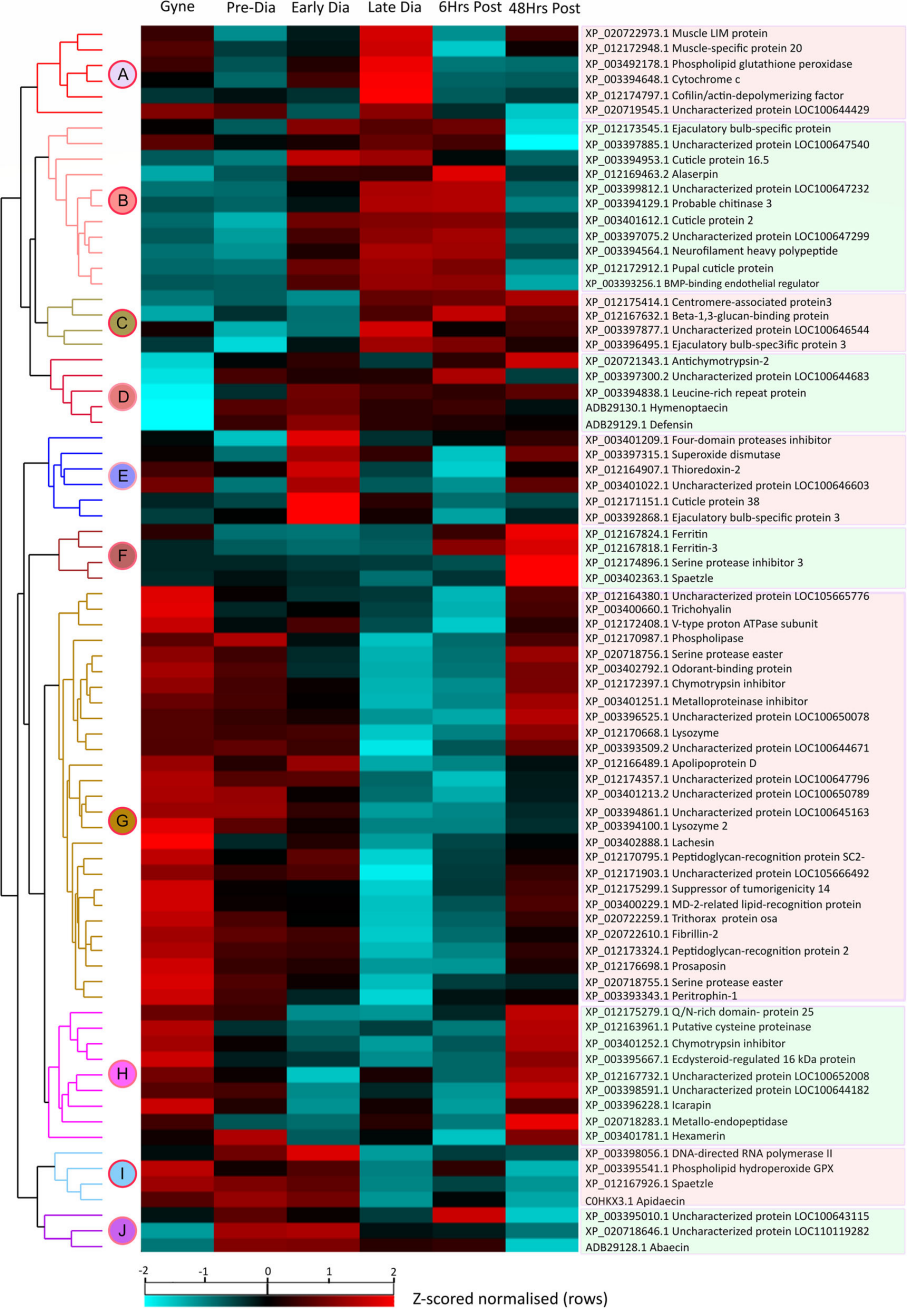
Distinct haemolymph proteome profile revealed during diapause. Two-way unsupervised hierarchical clustering of the median Z-score normalised label-free quantification (LFQ) intensity values of all statistically significant differentially abundant (SSDA) proteins (n = 79) for each life stage. Hierarchical clustering resolved 10 distinct clusters across the six queen life stages based on similar expression profiles of individual proteins. Differences in protein abundance are indicated by colour changes from low (green) to high (red) protein abundance representative of changes in Z-score normalised log2-fold transformed LFQ intensity values

To determine whether samples clustered based on life-cycle stage, we performed a principal component analysis (PCA) on Z-score normalised label-free quantification (LFQ) intensities for the 79 SSDA proteins. The first two principal components explained 29.6 and 18.19% of the variance within the dataset, respectively. The first principal component clearly separated early, late diapause and 6 h post-diapause individuals from the three other time-points (Fig. 1) highlighting distinct differences in the haemolymph proteome profiles associated with diapause. As a complementary approach, we performed hierarchical clustering of Z-score normalised LFQ intensities for the 79 SSDA proteins and identified ten distinct clusters of proteins with similar expression profiles (Fig. 2; Additional file 3: Table S3). Two clusters (Clusters B and C) comprise proteins that represent a clear diapause phenotype with proteins of high or highest abundance in the haemolymph of early diapause, late diapause or 6 h post-diapause queens. An additional cluster (Cluster A; *n* = 6) consisted of proteins that had the highest abundance during late diapause. There were also two clusters (Clusters G and H) of proteins with reduced abundances during diapause with the largest identified cluster (Cluster G; 27/79 proteins) consisting of proteins with low abundances during late diapause and six hours post-diapause. By 48 h post-diapause, the majority of these proteins had increased at least two-fold in abundance with levels comparable to pre-diapause haemolymph. Five proteins were identified with elevated abundances post-mating (Cluster D). Cluster E (*n* = 6 proteins) and cluster F (*n* = 4 proteins) comprised proteins with the highest abundances in early diapausing queens and 48 h post-diapause, respectively. An additional cluster (Cluster I; *n* = 4 proteins) consisted of proteins that had relatively high abundances in gynes, pre- and early-diapausing queens but decreased abundance in the haemolymph of late diapausing queens and at 48 h post-diapause. The last cluster (Cluster J; *n* = 3 proteins) consisted of proteins elevated post-mating and maintained at high levels during diapause before a reduction post-diapause.

### Comparative analyses of consecutive stages of the queen life cycle

Pairwise t-tests (*p* < 0.05) were performed to identify differences in mean LFQ intensities between consecutive time-points which enabled the characterisation of stage-specific changes in the queen life-cycle, including changes in response to mating, diapause entry, as well as diapause termination (Additional file 2: Table S2b).

#### Comparison of virgin and mated pre-diapause queens: increased abundance of AMPs in mated queen haemolymph

**Fig. 3:**
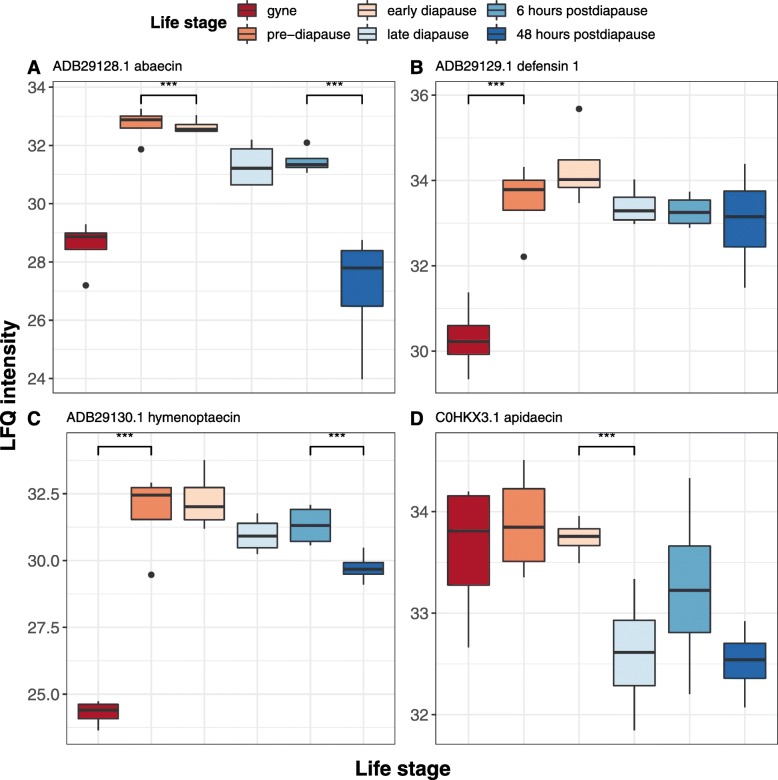
Elevated abundances of antimicrobial peptides in the queen haemolymph post-mating. Box plot representations of label-free quantification (LFQ) intensity values at six points throughout the bumblebee queen life-cycle for: (**a**) abaecin; (**b**) defensin; (**c**) hymenoptaecin; and (**d**) apidaecin. For each protein, the y-axis shows the LFQ intensity values representative of protein abundance. The x-axis represents the six sampled queen life stages. Each box is coloured to indicate a different life stage of the queen. Stages with a significant difference in abundance (two-sample t-test; *p* < 0.05) are indicated with asterisks

We identified ten proteins with significant (two-sample t-test; *p* < 0.05) abundance changes between the haemolymph proteome of virgin and mated queens (Additional file 2: Table S2b). Three of these proteins were annotated as putative antimicrobial peptides (AMPs) with the greatest increase evident for hymenoptaecin (ADB29130.1), which was 183-fold more abundant (*p* < 10^− 4^; Fig. 3) in the haemolymph of mated queens. Increased abundances were also observed for defensin (17.9 fold increase; *p* < 10^− 4^) and abaecin (9.4 fold increase; *p* < 10^− 3^; Fig. 3). Post-mated queens also had increased abundance of a putative esterase FE4 (XP_003397300.2; 7.24 fold increase; *p* < 10^− 2^) and a putative kappa-theraphotoxin-like protein (XP_020718646.1; 3.48 fold increase; *p* < 0.05). Five proteins had reduced abundances within the mated queen haemolymph including a BMP endothelial regulator protein (XP_003393207.1), an ecdysteroid regulated 16 kDa (XP_003395667.1), a ferritin subunit (XP_012167824.1), a putative muscle protein (XP_020722973.1) and an odorant binding protein (XP_003397877.1) demonstrating that mating can affect the expression of proteins involved in diverse biological processes.

#### Comparison of mated pre-diapause and early diapause queens: early diapause phenotype characterised by an increase in cuticular and ejaculatory bulb-specific proteins

**Fig. 4:**
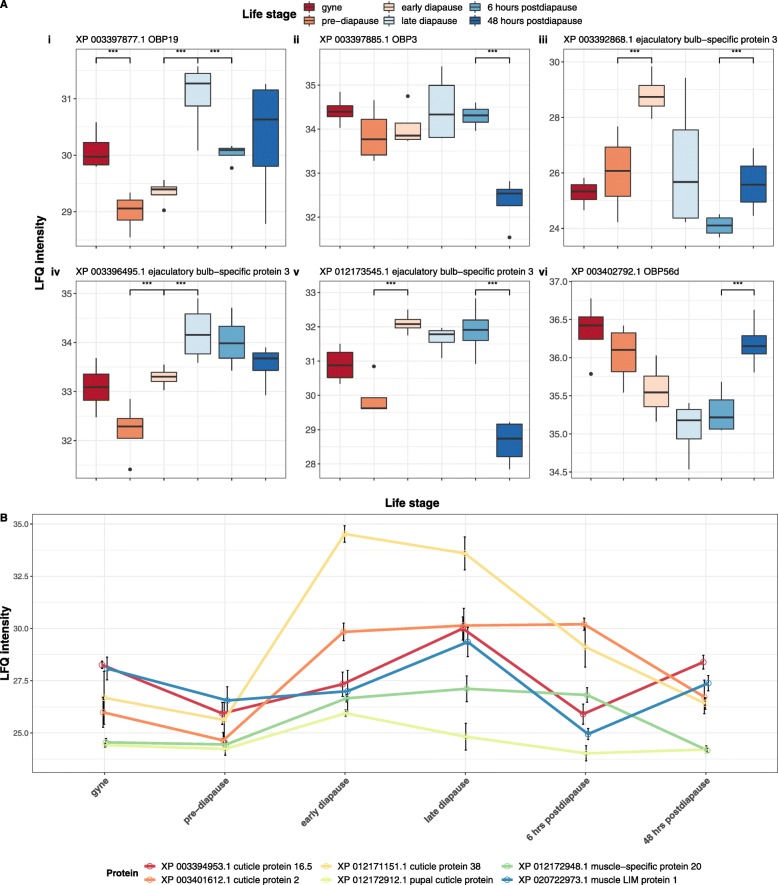
Olfaction and structural-related protein changes in queen haemolymph in response to diapause. **a** Box plot representations of protein abundances at six points throughout the bumblebee queen life-cycle for six olfaction-related proteins including three odorant binding proteins and three ejaculatory bulb-specific proteins. For each protein, the y-axis shows the LFQ intensity values. The x-axis represents the queen life stages. Each box is coloured to indicate a different life stage of the queen. Stages with a significant difference in abundance (*p* < 0.05) are indicated with astrisks. **b** Line plot shows relative abundance changes between structural-related proteins within the queen haemolymph at six time-points. The y-axis shows the LFQ intensity values. The x-axis represents the individual queen life stages

Sixteen proteins changed significantly in abundance between the haemolymph of mated pre-diapause and early diapause queens. There was a significant trend of elevated expression of proteins (*n* = 13) in the haemolymph of early diapause queens (binomial test, *p* = 0.02). Of the 13 proteins with elevated abundance, three were annotated as ejaculatory-bulb specific proteins (each had at least > 2 fold increase; *p* < 0.05; Fig. 4)a, while four were annotated as cuticular proteins, including one protein which was increased 478-fold within the haemolymph of early diapause queens in comparison to pre-diapause queens (Fig. 4)b. Additional proteins with significantly increased abundances included a serine protease inhibitor (XP_003401209.1), a neurofilament heavy polypeptide (XP_003394564.1), a heat shock protein (XP_003402976.1), a gamma interferon-inducible lysosomal thiol (GILT) reductase (XP_003397075.2), a putative scavenger receptor protein (XP_012163150.1) and a BMP-binding endothelial regulator (XP_003393256.1). We identified three proteins with significant reductions in the early diapause queen haemolymph, including a putative salivary protein (XP_012167732.1), a prion-like protein (XP_012175279.1) and a hexamerin (XP_003401781.1), which was reduced 23.9 fold.

#### Comparison of early and late diapause queens: selective reduction in immune expression in late diapause queen haemolymph

We identified 29 SSDA proteins between the haemolymph of early and late diapause queens (Additional file 2: Table S2b). The majority of these proteins (*n* = 21) had lower abundance within late diapause queens, which was a significant trend (binomial test, *p* < 0.03). Of the 21 proteins whose abundance was reduced at least 1.5 fold in the haemolymph of late diapause queens, 12 had putative roles in immunity, including two pathogen recognition proteins, two immune signalling proteins, five effector proteins, two regulatory proteins and one putative detoxification enzyme.

#### Comparison of late diapause and 6 h post-diapause queens: reduced abundance of detoxification and structural proteins in queen haemolymph post-diapause

We identified 14 SSDA proteins within the queen haemolymph between six hours post-diapause and late diapause (Additional file 2: Table S2b). Of the nine proteins significantly reduced at least 2 fold within the haemolymph of six hours post-diapause queen haemolymph, three proteins were annotated with putative roles in detoxification (XP_003397315.1: superoxide dismutase; XP_020719829.1: glutathione peroxidase; XP_003394648.1: cytochrome c). Four of the reduced proteins had putative structural roles with two annotated as muscle-associated proteins (XP_012172948.1; XP_020722973.1), one annotated as an actin-depolymerising protein (XP_012174797.1), and one protein annotated as a cuticular protein (XP_003394953.1). The final two proteins with significantly reduced expression were annotated as an odorant binding protein (XP_003397877.1) and pterin-4-alpha-carbinolamine dehydratase (XP_012170199.1). Five proteins had at least a 1.3 fold increase in abundance, including an endoribonuclease (XP_012167789.1), a glyoxylate reductase/hydroxypyruvate reductase (XP_012170485.1), an iron-binding ferritin (XP_012167818.1), a putative clotting factor (XP_012175299.1), as well as a hypothetical protein of unknown function (XP_003395010.1).

#### Comparison of 6 and 48 h post-diapause queens: recovery in queen reproductive and immune protein expression at 48 h post-diapause

The greatest number of SSDA proteins (*n* = 52) was identified between the haemolymph proteome of queens at 6 h and 48 h post-diapause with the majority (*n* = 32) increasing in abundance in the haemolymph 48 h post-diapause although this pattern was not significant (binomial test, *p* = 0.1263). The greatest increase in abundance was seen in a storage protein (XP_003401781.1: hexamerin), which had a 49.8 fold increase. Two other putative reproductive proteins (XP_020718283.1: membrane metalloendopeptidase-like 1; XP_012163499.1: vitellogenin) increased in abundance at least 5-fold in the haemolymph at 48 h post-diapause. The haemolymph proteome also had increased abundances of proteins with putative roles in immunity (*n* = 12), muscle-associated proteins (*n* = 2), olfaction (*n* = 2), venom (*n* = 3), as well as proteins of unknown function (*n* = 5). The remaining proteins were annotated with putative roles in cholesterol metabolism (XP_003395667.1: ecdysteroid-regulated 16 kDa), chitin binding (XP_003393340.1: peritrophin-1), neurotransmitter synthesis (XP_003401022.1: glutamate decarboxylase), as well as gene expression regulation (XP_020722259.1: trithorax).

Twenty proteins had significantly reduced expression in the queen haemolymph at 48 h post-diapause. The protein with the greatest reduction was a single domain Von Willebrand factor type C domain-containing protein (XP_003399812.1), which had a 22-fold reduction. Certain immune-associated proteins, including two AMPs (ADB29130.1: hymenoptaecin; ADB29128.1: abaecin), two putative serine protease inhibitors (XP_003398424.1: serpin-B3; XP_012169463.2: alaserpin), and a putative anti-viral protein (XP_003399869.1: protein son of sevenless**)** were reduced in comparison to the haemolymph of queens at six hours post-diapause. A sixth putative immune protein, a leucine-rich repeat domain-containing protein (XP_003394691.1) also had reduced abundance. Five proteins with putative structural roles were reduced, including three cuticular proteins, a neurofilament heavy polypeptide (XP_003394564.1) and a chitinase (XP_003394129.1). Similar to other time-points, putative olfaction-related proteins were also affected with two proteins (XP_003397885.1: OBP3; XP_012173545.1: PEBIII) reduced within the haemolymph at 48 h post-diapause. Other proteins reduced at least 2.6 fold included a glyoxylate reductase/hydroxypyruvate reductase (XP_012170485.1), a GILT protein (XP_003397075.2), an esterase (XP_003397300.2), a glutathione peroxidase (XP_003395541.1), a protein of unknown function (XP_003395010.1) and a putative BMP-binding endothelial regulator (XP_003393256.1). Interestingly, this last protein is coded for by a gene that forms part of a novel gene family consisting of five genes coding for proteins expressed at high abundance in the queen haemolymph (Additional file 4; Additional file 2: Table S2b).

### Proteogenomic analysis of a novel haemolymph-associated protein family

It is estimated that more than 40% of genes in sequenced eukaryotic genomes do not have assigned functions [64]. In our analysis, we provide evidence for the expression of 33 hypothetical or uncharacterised proteins in the queen haemolymph, of which 16 changed in abundance over the life stages of the queen. Of most interest, we identified five novel proteins which shared high sequence similarity (minimum sequence similarity of 45.75%; E-value <1e-58) that were all expressed in the haemolymph proteome. These five proteins were coded for by four individual protein-coding genes suggestive of a previously undocumented gene family (Additional file 5: Table S4).

These four genes, hereafter known as the highly abundant haemolymph-associated protein (HAHP) family are located within a genomic region spanning approximately 100 kb on chromosome one (BG_1; NC_015762.1) in the *B. terrestris* genome (Additional file 4: Fig. S1). There is evidence of a fifth protein-coding gene (LOC110119444), but this predicted protein was not identified in the queen haemolymph in our analysis. Although one of the proteins was annotated as a BMP-binding endothelial regulator protein (XP_003393256.1), additional functional domain analysis did not identify any conserved domains present on any of the HAHP family members. At the nucleotide and protein level, we identified high sequence similarity with five putative orthologues identified in the genome of the Eastern bumblebee, *B. impatiens*. We performed homology searches against other insect predicted proteomes identifying single protein matches in each of the four sequenced honeybee (*Apis*) genomes. Similarly, functional domain analysis of honeybee species homologues only identified the presence of a predicted signal peptide domain. No homologous sequence was identified for the fruit fly *Drosophila melanogaster*.

Interestingly, one member of the HAHP family (XP_003393256.1; LOC100652150: HAHP1) displayed an expression profile that suggests a potential role in diapause (Additional file 4: Fig. S2). This protein has relatively high abundance levels in gynes and pre-diapause queens and significantly increases in early, mid diapause and 6 h post-diapause queens. Protein abundance returns to pre-diapause levels at 48 h post-diapause.

### Weak conservation of protein and transcript expression profiles during diapause

In an attempt to explore the conservation of molecular mechanisms underlying diapause, we compared our proteomic dataset with a previously published transcriptomic dataset of bumblebee queens collected before, during and after diapause [46]. We identified 114 genes (88.4% of genes coding for haemolymph-associated proteins) expressed in the queen fat body at three time-points [46](Additional file 6: Table S5). Of this number, only three genes (LOC100643414: protein spaetzle; LOC100648549: cytochrome c; LOC100651094: glycine-rich cell wall structural protein) had conserved directional changes in transcript and protein abundance during diapause (Additional file 4). The majority differed in direction of expression profile at the transcript and protein level, which was a significant trend (binomial test, *p* < 0.005) and highlights a weak association between transcript and protein expression. We identified two Gene Ontology terms (GO:0032504, ‘multicellular organism reproduction’; GO:0005615, ‘extracellular space’) as significantly enriched in both the transcriptome and haemolymph proteome (Additional file 7: Table S6).

## Discussion

Diapause is a key component of insect life-cycles. Using bumblebee queens as a model system to investigate physiological adaptations to diapause, we show that: 1) mating is followed by significant increases in antimicrobial peptides, which are maintained at highly elevated levels throughout diapause; 2) in contrast to elevated AMP expression, other immune proteins reduce in abundance during diapause; 3) diapause is also characterised by increases in cuticular and muscular proteins, and 4) due to weak conservation in transcript and protein expression, transcriptomic approaches alone may not fully represent the biology of diapause phenotypes. Taken together these results provide novel insights into the molecular mechanisms involved in diapause preparation, maintenance and termination within an ecologically and economically important pollinator.

**Fig. 5:**
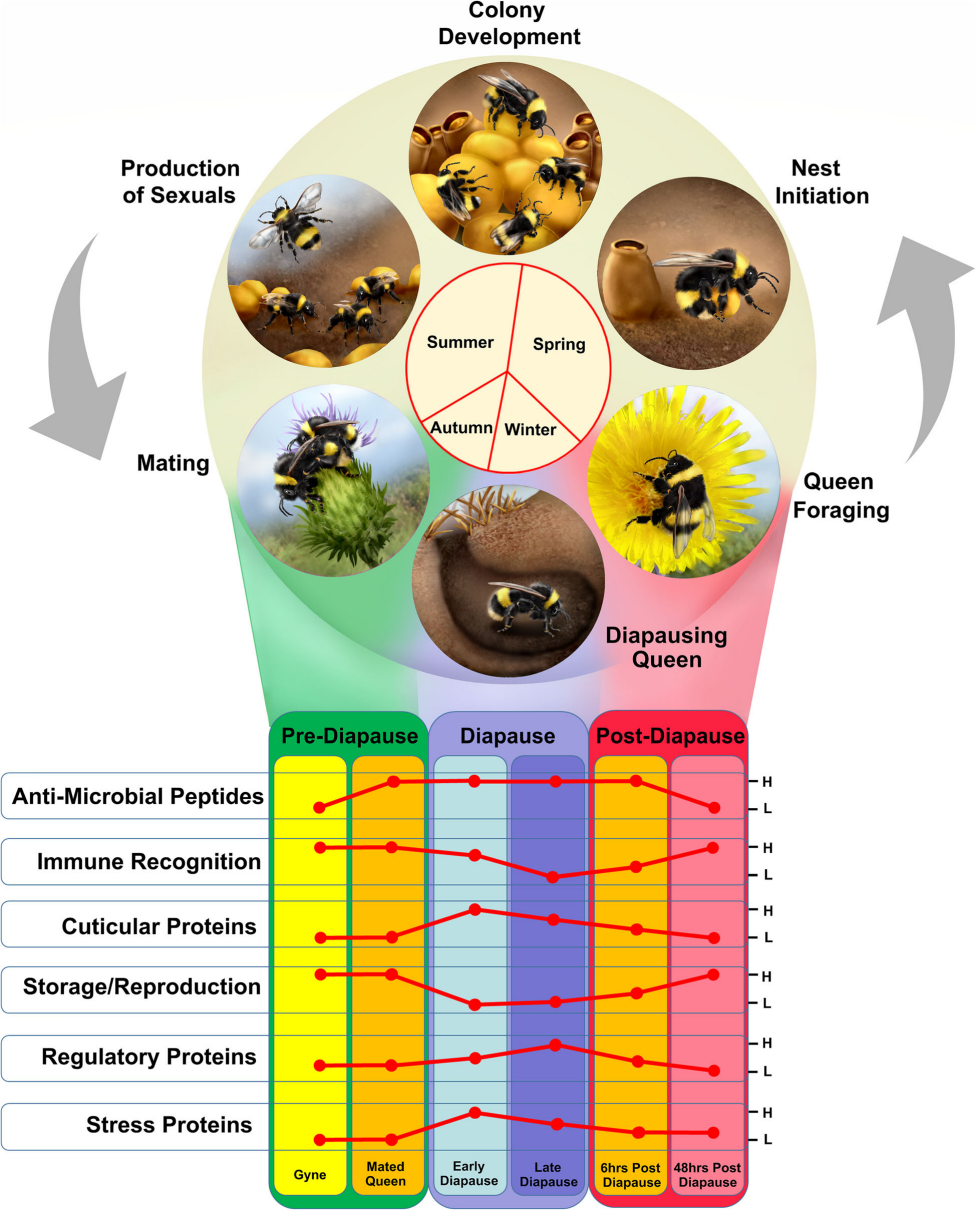
Summary overview of protein expression profiles in relation to life stage and colony life cycle. Broad trends in the abundance of protein categories in unmated and mated queens prior to (green), during (blue) and after (red) diapause. Proteins associated with the immune system, cuticle, storage and reproduction, regulation and stress displayed characteristic expression profiles across diapause suggestive in roles in the preparation for, maintenance of and recovery from diapause. L and H represent low and high relative protein abundances, respectively

Dormancy strategies, such as diapause, have evolved to allow organisms avoid periods of harsh environmental conditions when resources may be low [3, 65]. A characteristic of diapause is an arrest in development and reproduction. These processes are metabolically and energetically demanding, which can result in physiological and evolutionary trade-offs with other biological processes [66]. While oogenesis places energetic demands on the adult female [67], the act of mating can result in behavioural and physiological changes in the female phenotype [66]. While the arrest of reproductive potential may free up resources for other costly physiological processes, such as immunity, this has not been previously investigated during diapause. We identified dynamic changes in protein profile and abundance during diapause (summarised as Fig. 5), especially in relation to immunity-associated proteins. Firstly, mating was followed by significant increases in the abundance of antimicrobial peptides, including a defensin, a hymenoptaecin and an abaecin in the haemolymph. Antimicrobial peptides are generally induced upon infection and display antibacterial and antifungal activities including inhibition of cell growth and lytic activity of foreign microorganisms [68–71]. While mating can result in expression of AMPs in the female reproductive tract possibly to restrict the movement of sexually transmitted diseases, copulation in insects generally results in reduction in female immune potential [66]. This immunosuppression is suggested to be due to resources being used to increase fecundity, which is regulated by products of the endocrine system, which promote reproductive development and suppress aspects of the immune system, including expression of AMPs. However, in females that undergo diapause, organs that synthesise and secrete hormones that regulate reproductive development, such as juvenile hormone (JH), are not fully developed [72]. The lack of development of the corpora allata may be the reason for both increased expression at the protein and transcript level in bumblebee queens. Furthermore, increased AMP expression in mated queens is associated with increased resistance to the trypanosome, *Crithidia bombi*, demonstrating the immune benefits of mating [43]. Here, not only do we provide evidence of elevated protein abundances post-mating but also show that these levels are sustained during diapause for a period up to 120 days (approximately one-third of a queen’s expected lifespan). Given how energetically costly activation and constitutive immune expression is for an organism [15, 73], the maintenance of elevated protein levels in the haemolymph would be suggested to directly increase diapause survival rather than as an indirect consequence of mating. Indeed, infections can occur during and immediately after diapause [44, 74, 75], which can affect survival and therefore, immune defences are required. However, selective expression of immune components may be sufficient to reduce infections.

In contrast to AMP expression, we identified a reduction in proteins with putative immunological roles. Periods of reduced nutritional intake, such as hibernation and diapause, can reduce resources available for certain immune expression and activity. Even in non-immune challenged individuals, metabolic trade-offs are evident between different facets of the immune response, most notably between the humoral and cellular response [76, 77], with different nutritional demands suggested for the expression of each arm [78]. The reduction in immune proteins observed in our study may also be self-regulatory, as suggested by the increase in serine protease inhibitors, proteins which reduce and inactivate serine proteases, during queen diapause. Increased expression of AMPs, such as *drosomycin* and *cecropin*, has been seen in diapausing *D. melanogaster* adults in response to bacterial infection [13]. In the same study, a *peptidoglycan recognition protein* (*PGRP*) was not elevated in diapause samples in comparison to post-diapause samples providing additional evidence that aspects of the host immune system display differences in expression during diapause. Overall, we suggest that sentinel and responsive components of the immune system are depressed during diapause when the metabolic costs of driving these systems may not be sustainable. This view is supported by the fact that the end products of these recognition and signalling pathways are AMPs, which are highly elevated throughout diapause. As such, an anticipatory or prophylactic defence system may have evolved in bumblebee queens to protect the diapausing queen from microbial attack without the need to first detect and respond to pathogen presence.

While infections can reduce rates of diapause survival, diapause also represents a period where organisms are at risk of desiccation and adaptations are required to reduce water loss. One important barrier to water loss is the cuticle [79]. During the early diapause stage, cuticular proteins had elevated abundances in the queen haemolymph. Previously, a cuticular protein identified in the head tissues of early diapause *Culex* adult females [80] was suggested to function in cuticle reinforcement during this period. Increased protein expression in the haemolymph of both *Bombus* and *Culex* suggests a putative conserved role of cuticular proteins in diapause. While the exact function of these proteins are uncharacterised, the expression of cuticular proteins, which function in cuticular development during metamorphosis, is regulated by ecdysteroids, such as JH [81]. Consequently, low levels of JH [45, 46] evident during adult diapause for queens and other insects may facilitate the elevated expression of cuticular proteins providing an important defence against desiccation.

The diapause phenotype is tightly regulated by hormones affecting numerous biological processes of the diapausing individuals [82]. Our study has determined that such processes may involve the expression of olfaction-related proteins, most notably odorant binding proteins (OBPs) and ejaculatory bulb-specific proteins (PEBs), which display complex expression changes in queen haemolymph. OBPs and PEBs represent families of small hydrophilic molecules, which have been associated with the transportation of odour molecules in insect haemolymph [83, 84]. While the association of these proteins with olfactory organs, such as antennae [85], has suggested a role in olfaction, additional roles in other processes have been identified, including reproduction and immunity. For example, ejaculatory bulb-specific proteins, which contain chemosensory protein domains, have been characterised as major components of ejaculatory bulbs in the fruit fly *D. melanogaster* [86, 87]. While the exact role of these proteins in *B. terrestris* queens will require experimental validation, these types of proteins have also been shown to increase in abundance in flies in response to viral infection, although abundance did not coincide with a reduction in pathology suggesting indirect roles in viral response, such as tissue repair or behavioural response to infection [88]. Interestingly, this study also identified suppression of *PEBIII* expression in S2 cells exposed to the moulting steroid hormone, 20-hydroxyecdysone (20E), an ecdysteroid also involved in eliciting diapause termination within a number of insect species [88]. Similarly, the expression of OBPs in the developing antennae of the tobacco hawkmoth *Manduca sexta* is regulated by declining levels of the ecdysteroid 20E [89]. We identified the abundance of three OBPs to be affected during diapause although there was no general pattern of their response to diapause with only an OBP19 having elevated expression during early and late diapause suggestive of a potential role in diapause maintenance. While 20E ecdysteroid levels were not examined in the present study, low ecdysteroid levels have been found in bumblebee queens during diapause [46]. Thus, changes in their abundance in the haemolymph may be a consequence of changes in ecdysteroid levels associated with insect diapause. Further work will be required to elucidate the specific roles performed by olfaction-associated haemolymph proteins during insect diapause.

The convergence of diapause phenotypes across phylogenetically diverse taxa has led to the suggestion that a conserved genetic toolkit may regulate diapause [46, 63, 90]. To explore conserved changes at the transcript and protein level in queen diapause, we compared changes in the haemolymph proteome to transcriptional changes identified by a previous study in the fat body of bumblebee queens [46]. The insect fat body has an important role in storage and energy usage, as well as playing a central role in biosynthesis and metabolic activity [91], and also synthesises most of the haemolymph proteins [92]. Due to this role in haemolymph protein synthesis, it was unsurprising to find that the majority of haemolymph-associated proteins were transcribed in the fat body across three time-points analysed by Amsalem et al. [46]. However, we identified only weak overlap in expression profiles between the fat body transcriptome and haemolymph proteome. While transcriptomes provide important insights into genome-wide transcriptional regulation, here we show that these signals may not translate at the protein level, which is an important finding when considering candidate genes involved in the expression of the diapause phenotype. While these differences may be explained by biological and technical differences between the studies [93], a similarly modest correlation between mRNA transcript and protein levels has been described within model organisms, such as mice [94] and humans [95]. The limited overlap between the transcriptome and proteome highlights that in certain cases gene expression may not be an appropriate proxy for protein products expressed during diapause.

Given that the haemolymph represents a non-cellular fraction, proteomics has an added advantage of providing direct information on spatial and temporal protein expression and potential function not amenable by RNA-directed methods. For example, our results supplement recent genomic resources for bumblebee species [96–99], through the provision of translational and spatial evidence for 129 multiple-peptide supported haemolymph-associated proteins, including seven uncharacterised proteins devoid of known functional motifs and several members of a putative expanded gene family in queen haemolymph. Previous work by Sadd et al. [92] described 17 haemolymph-associated proteins of unknown function in post-diapause queen haemolymph (HAP1–14 and three putative members of the HAHP family). Three of these HAHP members, including an additional fourth, expressed in the haemolymph throughout the queen life cycle were identified in this study. In addition, the abundance of one member (HAHP1) changed during diapause suggesting a potential role in diapause maintenance. The HAHPs share sequence similarity with homologous proteins annotated as BMP endothelial regulator proteins and kielin/chordin-like proteins, which is suggestive of a role in development. However, their exact role in bumblebees will require further genomic, functional and expression studies to elucidate the biological processes associated with this diverse, haemolymph profuse and potentially important group of proteins.

## Conclusions

Here we provide a novel insight into the molecular phenotype underlying insect diapause, identifying distinct haemolymph profiles associated with each of the key ecophysiological stages of diapause. Our data indicate that mating precedes a specific increase in antimicrobial peptides, which persists throughout queen diapause suggesting a key role for these proteins in the protection and survival of queens. We provide new perspectives on the molecular mechanisms underlying diapause, highlighting the increase in cuticular proteins to reduce desiccation, the reduction in non-AMP immune components, as well as highlighting dynamic changes in aspects of olfactory proteins throughout diapause. We also demonstrate a lag period in recovery post-diapause. Finally, our results highlight the importance of proteomics in understanding the molecular mechanisms underlying diapause regulation and maintenance. Future research in this area will benefit through increased tissue-specific profiling, as well as functional characterisation of haemolymph-associated proteins, to provide a greater understanding into the molecular mechanisms underlying diapause in bumblebees.

## Methods

### Animal handling and haemolymph collections

Four week old queen-right *Bombus terrestris audax* colonies were obtained from a commercial supplier (Koppert, the Netherlands) and maintained in an environmentally controlled room (27 ± 1 °C, 45% relative humidity (RH)) under red light illumination. Colonies were provided with pollen and sugar water (ApiInvert) *ad libitum* and maintained for approximately six weeks until new sexuals were produced.

For the purpose of characterising changes in the haemolymph proteome over life cycle stages related to diapause, six time-points were examined: 1) virgin queens (gynes); 2) pre-diapause mated queens; 3) early diapause queens; 4) late diapause queens; and post-diapause queens collected at 5) 6 h post-diapause; and 6) 48 h post-diapause. For each time-point, we collected haemolymph from four individual queens. In brief, each natal colony was checked twice daily for the presence of newly emerged gynes. Callow gynes detected within a 24 h period were transferred from the natal colony to a separate wooden box (22 × 15 × 10 cm) where they were supplied with sugar water (50%) and pollen *ad* *libitum.* Gynes were maintained in groups in these nurseries until 5 days post-eclosion, at which point we collected haemolymph from four individual virgin queens. On the same day, remaining sexually mature gynes were mated. Matings were performed under standard laboratory conditions [100] and queens were mated with males from different colonies to avoid full-sibling inbreeding [101]. Three days post-mating, we collected haemolymph from four newly mated queens. The remaining queens were transferred to individual 50 ml centrifuge tubes, which contained 5 ml autoclaved sand. Queens were entered into diapause via incubation of animals at 4 °C (RH of 70%) in the dark. Diapausing queens (*n* = 4) were sampled at seven days into diapause to examine proteomic changes associated with early diapause. We also collected a second time-point (*n *= 4) at 14 weeks into diapause (late diapause). Post-diapause, queens were transferred to a temperature-controlled room at 27 ± 1 °C (RH of 45%). Each queen was maintained in an individual plastic box (dimensions: (L) 11.5 cm; (W) 9 cm; (H) 7 cm) lined with autoclaved clean sand. We collected haemolymph from individual bees at six (*n* = 4) and 48 (*n* = 4) hours post-diapause, respectively.

For each haemolymph collection, queens were anaesthetized using carbon dioxide (CO_2_) (BOC, Ireland) and an incision was performed between the third and fourth sternite. Using this newly made incision, we collected haemolymph using a microcapillary tube and transferred the collected neat haemolymph into a fresh 1.5 ml collection tube containing 100 μl autoclaved phosphate buffered saline (PBS) (Sigma, Ireland). From each bee, we collected approximately 30 μl of neat haemolymph. We centrifuged this solution at 8000 x g at 4 °C for 5 min to pellet down haemocytes and other cellular debris. We transferred the supernatant to a fresh 1.5 ml collection tube and stored at − 80 °C for later use.

### Mass spectrometry and data analysis

Haemolymph samples were removed from − 80 °C storage, thawed on ice and biological impurities removed from the samples using the 2-D clean-up kit (GE Healthcare, UK), following the manufacturer’s instructions. The resultant protein pellets were resuspended in 300 μl 50 mM ammonium bicarbonate (Ambic), 1 mM calcium chloride (CaCl_2_). Protein abundance was then quantified using the Qubit fluorometer with the Qubit protein assay kit (Invitrogen, Ireland). We removed 50 μg from each sample and reduced each with 4 μl of 200 mM dithiothreitol (DTT) (Fisher Scientific, Ireland). We then heated reduced samples to 95 °C for 15 min followed by acetylation with 1 M iodoacetamide (IAA) and incubated at 25 °C for 45 min. The acetylation reaction was stopped by adding 20 μl of 200 mM DTT followed by incubation of the samples at 25 °C for 45 min. We digested 10 μg of reduced and acetylated proteins with 0.5 μl of 0.5 μg/μl trypsin (Medical Supply Company, Ireland) through incubation at 37 °C overnight. The samples were then speed vacuumed and stored at 4 °C once dry.

Dried peptide samples were resolubilised in 0.1% formic acid (Sigma Aldrich, Ireland) and subjected to liquid chromatography tandem mass spectrometry (LC-MS/MS) using a Thermo Scientific LTQ Orbitrap XL mass spectrometer connected to a Dionex Ultimate 3000 (RSLCnano) chromatography system. Peptides were separated by an increasing acetonitrile gradient on a Biobasic C18 Picofrit™ column (100 mm length, 75 mm ID), using a 60 min reverse phase gradient at a flow rate of 300 nL min^− 1^. All data were acquired with the mass spectrometer operating in automatic data-dependent switching mode. A high-resolution MS scan (300–2000 Da) was performed using the Orbitrap to select the seven most intense ions prior to MS/MS. For each scan performed, the twenty most intense ions were selected for MS/MS analysis.

Protein identification from the MS/MS data was performed using the Andromeda search engine in MaxQuant (version 1.5.6.5; http://maxquant.org/; [102]) to correlate the data against the protein reference sequences derived from the *B. terrestris* genome [96] obtained from the National Centre for Biotechnology Information (NCBI) repository (17,508 entries, downloaded September 2018). The following search parameters were used: first search peptide tolerance of 20 ppm (parts per million), second search peptide tolerance of 4.5 ppm (standard parameters for Orbitrap data) with cysteine carbamidomethylation as a fixed modification and N-acetylation of protein, oxidation of methionine as variable modifications and a maximum of two missed cleavage sites allowed, all standard. False discovery rates (FDR) were set to 1% for both peptides and proteins and the FDR was estimated following searches against a target-decoy database. Peptides with a minimum length of seven amino acids were considered for identification and proteins were only considered identified when more than one unique peptide for each protein was observed or when a single peptide was identified across multiple samples by multiple MS/MS spectra. The MS proteomics data and MaxQuant search output files have been deposited to the ProteomeXchange Consortium [103] via the PRIDE partner repository with the dataset identifier PXD014384.

Label-free quantification (LFQ) intensities were calculated using the MaxLFQ algorithm [104] from razor and unique peptides with a minimum ratio count of two peptides across samples, log_2_-transformed and replicates were assigned to their respective samples. Only proteins found in all four replicates in at least one life stage group were retained in the analysis. Where proteins were absent in one or more replicates of the other groups a data-imputation step was conducted to replace non-detected values with values that simulate signals of low abundant proteins. These imputed values were chosen randomly from the normal distribution of all LFQ intensities for each sample and specified by a downshift of 1.8 times the mean standard deviation (SD) of all measured values and a width of 0.1 times this SD.

### Statistical analysis to identify differentially abundant haemolymph proteins

Using Perseus (v. 1.5.5.3 [105]), we performed an analysis of variance (ANOVA) for multiple samples across the six life stage groups using a permutation-based false discovery rate (FDR) of 5% and below to indicate statistically significant differentially abundant (SSDA) proteins. The null hypothesis being tested was that there is no difference in haemolymph proteome prior to, in preparation for, during and after diapause. As a complementary measure to the analyses performed using Perseus, we performed a one-way ANOVA in R (v.3.5.1) using the LFQ intensity values. We identified a significant overlap in proteins detected as differentially abundant by the two approaches (binomial test, *p* < 10^− 10^, Additional file 4, Additional file 2: Table S2c) providing independent support for the findings of the analysis by Perseus. As the Perseus software package was specifically developed for the loading, imputation and analysis of proteomic data, we report only these results. Full details on the cross-validation are provided in Additional file 4 and the results are provided in Additional file 8: Table S7. A principal component analysis was performed using Z-score normalised LFQ intensity values for SSDA proteins and output visualised using ggfortify (v.0.4.5 [106]). Hierarchical clustering was performed using Perseus to produce a heatmap of protein abundance using Z-score normalised LFQ intensity values for all SSDA proteins by clustering both proteins using Euclidean distance and complete linkage.

To identify proteomic changes between each consecutive time-point of pre-, mid, and post-diapause samples, Student’s t-tests (*p* < 0.05) were performed by Perseus with the log2 difference between mean LFQ intensities obtained and used to determine relative abundance differences in individual proteins. Box and line plots for SSDA proteins were generated for specific protein groups to visualise their expression profile before, during and after diapause using ggplot2 (v. 3.1.0 [107]). Gene Ontology term enrichment for SSDA proteins was performed using a Fisher’s exact test (Benjamini Hochberg (BH) adjusted *p* < 0.05) with the ‘weight01’ algorithm (node size = 20) as part of the topGO package (v. 2.34.0 [108]).

### Analysis of a novel haemolymph-associated gene family

For each hypothetical protein identified within the haemolymph, we performed individual BLASTp [109] searches against the NCBI non-redundant (nr) database to identify putative homologues across taxonomically diverse groups to allow for inference of functional annotation. During these searches, we identified certain hypothetical proteins to share close sequence similarity (E-value threshold: 1e-40) to other hypothetical proteins suggestive that the proteins may be isoforms or coded for paralogous genes. To explore this further, we investigated the genomic coordinates of underlying genes to identify the proximity of putative paralogous genes to each other. Genomic coordinates of underlying protein-coding genes were visualised using arrow plots generated by gggenes (v.0.3.2).

### Transcript expression of haemolymph-associated proteins during diapause

Using data generated by Amsalem et al. [46], we quantified transcript expression, as well as differential expression of genes coding for haemolymph-associated proteins in the fat bodies of queens collected pre-, during and post-diapause. Additional information on the methods related to transcript quantification and differential expression is provided in Additional file 4. Scripts used for transcript quantification, differential expression and Gene Ontology enrichment analyses are modifications of the scripts published by Colgan et al. [110]. The modified scripts used in the present study are archived at https://github.com/Joscolgan/bumblebee_proteomics.

## Supplementary information

**Additional file 1: Table S1.** The 129 proteins identified from the haemolymph of unmated and mated queens prior to, during and after diapause.

**Additional file 2: Table S2.** Multivariate analysis (ANOVA FDR 5%) of all identified proteins from the haemolymph of unmated and mated queens prior to, during and after diapause.

**Additional file 3: Table S3.** All ANOVA statistically significant proteins (FDR < 0.05) used for hierarchical clustering.

**Additional file 4: Figure S1.** Genomic coordinates of members of a putative expanded haemolymph-associated gene family within the buff-tailed bumblebee genome. **Figure S2.** One HAHP family member increases in abundance during diapause. **Figure S3.** Gene Ontology term enrichment within significantly differentially abundant proteins in the bumblebee queen haemolymph proteome.

**Additional file 5: Table S4.** Genomic coordinates for members of a novel haemolymph-associated gene family.

**Additional file 6: Table S5**. Gene expression of the haemolymph-associated proteins in the bumblebee queen fat body.

**Additional file 7: Table S6.** Enriched Gene Ontology terms in genes coding for haemolymph-associated differentially expressed proteins during the bumblebee queen life cycle stages.

**Additional file 8: Table S7.** Pairwise t-tests were performed for each protein between consecutive life-cycle stages.

## Data Availability

Data are archived at PRIDE (PXD014384) and will be publicly available upon manuscript acceptance. Analytical scripts for gene expression analysis, Gene Ontology term enrichment and data visualisation are achieved on a public repository at https://github.com/Joscolgan/bumblebee_proteomics.

## Abbreviations

Ambic: Ammonium bicarbonate
AMP: Antimicrobial peptide
ANOVA: Analysis of variance
BMP: Bone morphogenetic protein
DTT: Dithiothreitol
FDR: False Discovery Rate
GILT: Gamma-interferon-inducible lysosomal thiol reductase
GO: Gene Ontology
HAHP: Highly abundant haemolymph-associated protein
IAA: Iodoacetamide
JH: Juvenile hormone
LC-MS/MS: Liquid chromatography tandem mass spectrometry
LFQ: Label-free quantification
NCBI: National Centre for Biotechnology Information
OBP: Odorant binding protein
PBS: Phosphate buffered saline
PCA: Principal component analysis
PEB: Ejaculatory bulb-specific protein
PGRP: Peptidoglycan recognition protein
ppm: Parts per million
RH: Relative humidity
SD: Standard Deviation
SSDA: Statistically significant differentially abundant
