# Supplementary material for: Mating precedes selective immune priming which is maintained throughout bumblebee queen diapause
Source: BMC Genomics. 2019 Dec 10;20:959. doi: 10.1186/s12864-019-6314-9 (PMC6902353; doi:10.1186/s12864-019-6314-9)
Supplement: Supplementary file 4 — Additional file 4: Figure S1. Genomic coordinates of members of a putative expanded haemolymph-associated gene family within the buff-tailed bumblebee genome. Figure S2. One HAHP family member increases in abundance during diapause. Figure S3. Gene Ontology term enrichment within significantly differentially abundant proteins in the bumblebee queen haemolymph proteome. [file 12864_2019_6314_MOESM4_ESM.pdf]

Supporting information for:

Mating precedes selective immune priming which is maintained throughout bumblebee queen diapause

Thomas J. Colgan, Sive Finlay, Mark J. F. Brown & James C. Carolan

Cross validation of Perseus results.

As a complementary measure, we performed a one-way ANOVA in R (v.3.5.1) using the label-free quantification (LFQ) intensity values, which identified 68 proteins as differentially abundant across the six time-points examined. All 68 proteins were also detected by the ANOVA employed by Perseus, which was a significant trend (binomial test, $p < 10^{-10}$) providing additional evidence that the abundance of these proteins change significantly throughout the queen life-cycle (Supporting Tables S2 and S7). Similarly, we also performed pairwise t-tests (Benjamini-Hochberg adjusted $p < 0.05$) between consecutive time-points using R identifying a total of 81 proteins as significantly differentially abundant (Supporting Tables S2 and S7). Sixty nine of these proteins were also detected to change between consecutive time-points through pairwise comparisons by Perseus (n = 79 proteins), which was also a significant trend (binomial test, $p < 10^{-11}$). These steps provided additional confidence in the use of proteins identified as significantly differentially abundant by Perseus.

Comparative analysis of haemolymph proteome and fat body transcriptome.

To identify conservation in transcript and protein expression during diapause, we obtained raw reads for 15 bumblebee queen fat body RNA-Seq libraries generated by Amsalem et al. [1] from the NCBI Short Read Archive (SRA) database (BioProject ID: PRJNA295976). These libraries were generated from queens collected before (n = 5), during (n = 5) and after diapause (n = 5). For transcript quantification and differential expression analyses, we used publicly available scripts published by Colgan et al. [2]. We aligned reads against a predicted transcriptome of *Bombus terrestris* [3] using the *k*-mer based probabilistic pseudoaligner kallisto (v. 0.45; [4]). We generated estimated gene-level counts using tximport [5], which we used as input for differential expression analysis using DESeq2 [6]. We implemented a likelihood ratio test (LRT) within DESeq2 to identify genes with significant changes (Benjamini Hochberg (BH) adjusted $p < 0.05$) in amplitude expression across three time-points (pre-, during and post-diapause). Furthermore,

we used the Wald test in DESeq2 to identify significant abundance changes (BH adjusted $p < 0.05$) between the pre and during diapause time-points but also between the during and post-diapause time-points. Using this approach, we identified 810 and 5,292 genes as differentially expressed between pre- and during diapause queens and diapausing and post-diapause queens, respectively. As a preliminary measure to compare transcript and protein expression during queen diapause, we investigated the qualitative expression of genes coding for haemolymph-associated proteins in the queen fat bodies. We identified 114 genes (88.4% of genes coding for haemolymph proteins) to be expressed in the queen fat bodies at all three time-points sequenced by Amsalem et al. [1] (Supporting Table S5).

Differential expression between pre- and late diapause queens.

To identify conserved changes in transcript and protein expression between pre-diapause and diapausing queens, we compared differentially expressed genes in the fat body with genes coding for proteins that changed significantly (two sample t-test $p < 0.05$) in abundance between pre-diapause and late diapause queens. We identified eight genes underlying haemolymph-associated proteins as significantly differentially expressed (BH-adjusted $p < 0.05$) between the fat bodies of pre-diapause mated and diapausing queens. The majority (n = 7) had reduced expression in the fat bodies of diapausing queens although this trend was not significant (binomial test, $p = 0.07$). Of these eight protein-coding genes, two genes had significant differential expression between mated pre-diapause and late diapause queens. The first gene (LOC100643414: protein spaetzle) had higher expression at both the transcript and protein level in pre-diapause mated queens in comparison to late diapause time-points. The second gene (LOC100645985: cuticle protein 16.5) had a more complex expression with significantly reduced transcript expression during diapause but elevated protein abundance in the diapausing queen haemolymph.

Differential expression between diapausing and post-diapause queens.

To identify genes with similar transcript and protein expression post-diapause, we compared genes differentially expressed between during diapause and post-diapause foundress queens with genes coding for haemolymph-associated proteins differentially abundant between late diapause and 48 hours post-diapause queens. We identified 57 genes coding for haemolymph-associated proteins as differentially expressed in the queen fat body between post-diapause foundress queens and queens collected during diapause. Similar to the

pre-diapause mated queens, the majority of the genes of interest (n = 40) had reduced expression during diapause, which was a significant trend (binomial test, $p < 0.004$). 16 of these genes had significant changes in protein abundance between diapausing and 48 hours post-diapause queens. Of this number, only two genes (LOC100648549: cytochrome c; LOC100651094: glycine-rich cell wall structural protein) had similar changes in transcript and protein abundance while the majority (n = 55) differed in direction of expression between the transcript and protein level, which was a significant trend (binomial test, $p < 0.005$).

Functional domain and Gene Ontology enrichment analysis.

Functional domain analysis annotated 79.8% (n = 103) of haemolymph-associated proteins with predicted signal peptide domains indicating that these proteins are secreted. Over half of the proteins (n = 70) were annotated with predicted protein domains with the most common domains being ‘chitin binding domain’ and ‘chitin domain superfamily domain‘. To identify biological processes affected during the queen life-cycle, we performed a Gene Ontology enrichment analysis on the SSDA proteins (n = 79). We identified 15 terms that were enriched for proteins identified as SSDA across the queen life-cycle stages, including ten associated with ‘biological processes’, three with ‘molecular function’ and two associated with ‘cellular process’ (Supporting Table S6; Supporting Figure S3). Of the ten GO enriched terms associated with ‘biological processes’, nine were associated with the immune response, which was a significant trend (binomial test, $p = 0.02$). The three most significant enriched immune-associated terms included ‘regulation of Toll signalling pathway’, ‘defense response to Gram-negative bacteria’, and ‘defense response to fungus’. Aside from immune-associated terms, the only other BP-associated GO term was ‘multicellular organism reproduction’ (Supporting Figure S3).

Gene Ontology Enrichment analysis of fat body transcriptome

As an additional measure, we examined Gene Ontology terms enriched within differentially expressed genes in the fat body transcriptome and differentially abundant proteins in the haemolymph proteome to identify conserved biological processes affected in both by diapause. Using the genes identified as significantly expressed across the three time-points (n = 6000; LRT, BH adjusted *p*-value < 0.05), we identified a total of seven Gene Ontology terms as significantly enriched (Fisher’s exact test: FDR < 0.05). We identified only two terms (GO:0032504, ‘multicellular_organism_reproduction’; GO:0005615, ‘extracellular_space’) as significant within both the transcriptome and haemolymph proteome (Supporting Table S6).

Supporting Figures

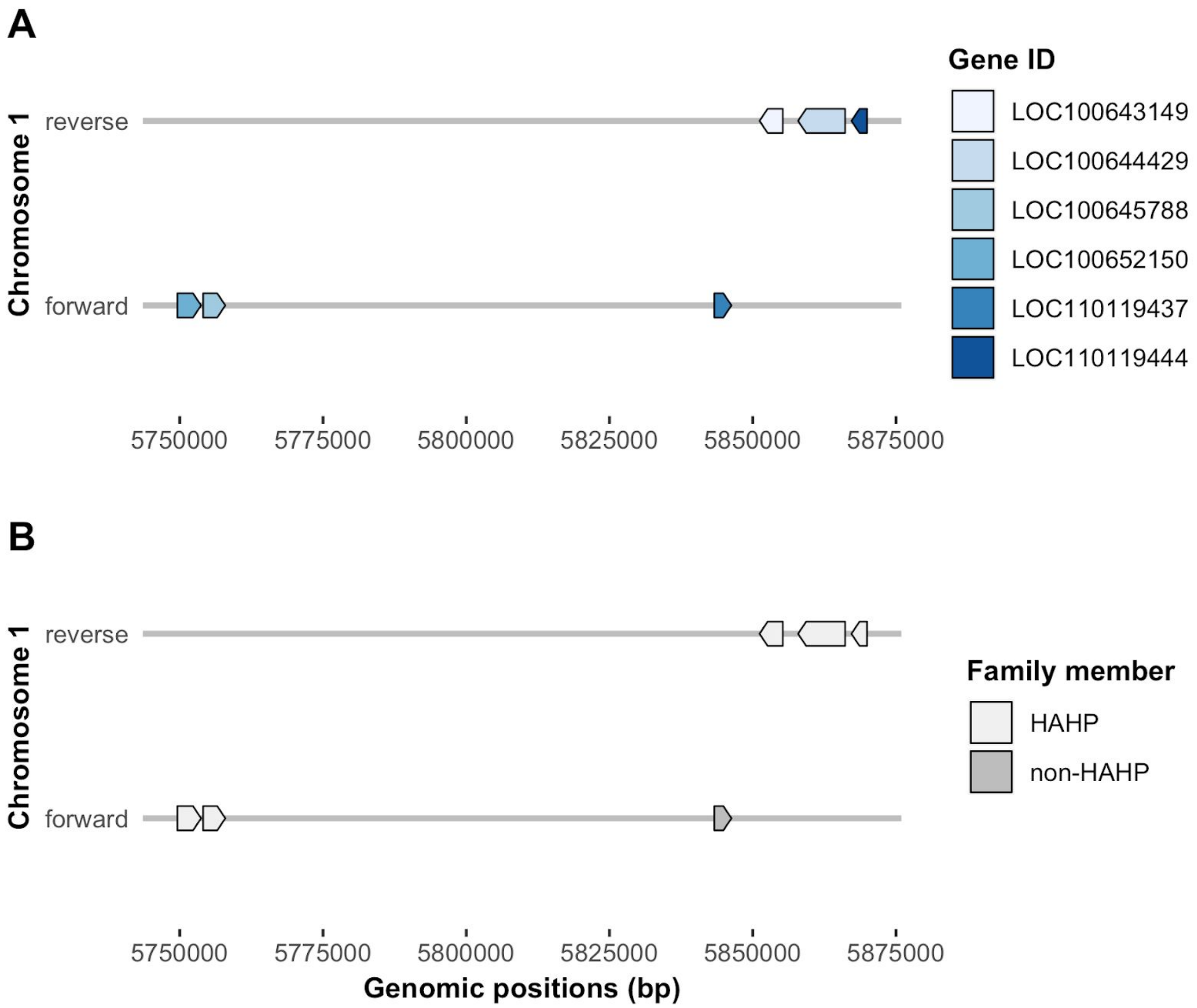

Figure S1. Genomic coordinates of members of a putative expanded haemolymph-associated gene family, within the buff-tailed bumblebee genome. (A) Arrow plot shows the genomic coordinates of members of the proposed highly abundant haemolymph-associated protein (HAHP) family. Each arrow represents an individual gene with the size of arrow relative to gene size. The direction of arrow indicates the strand orientation of each gene while each gene is represented by an individual shade of blue. (B) The second arrow plot indicates which of the six genes represent within this region are proposed members of the expanded HAHP family. HAHP members are coloured light grey.

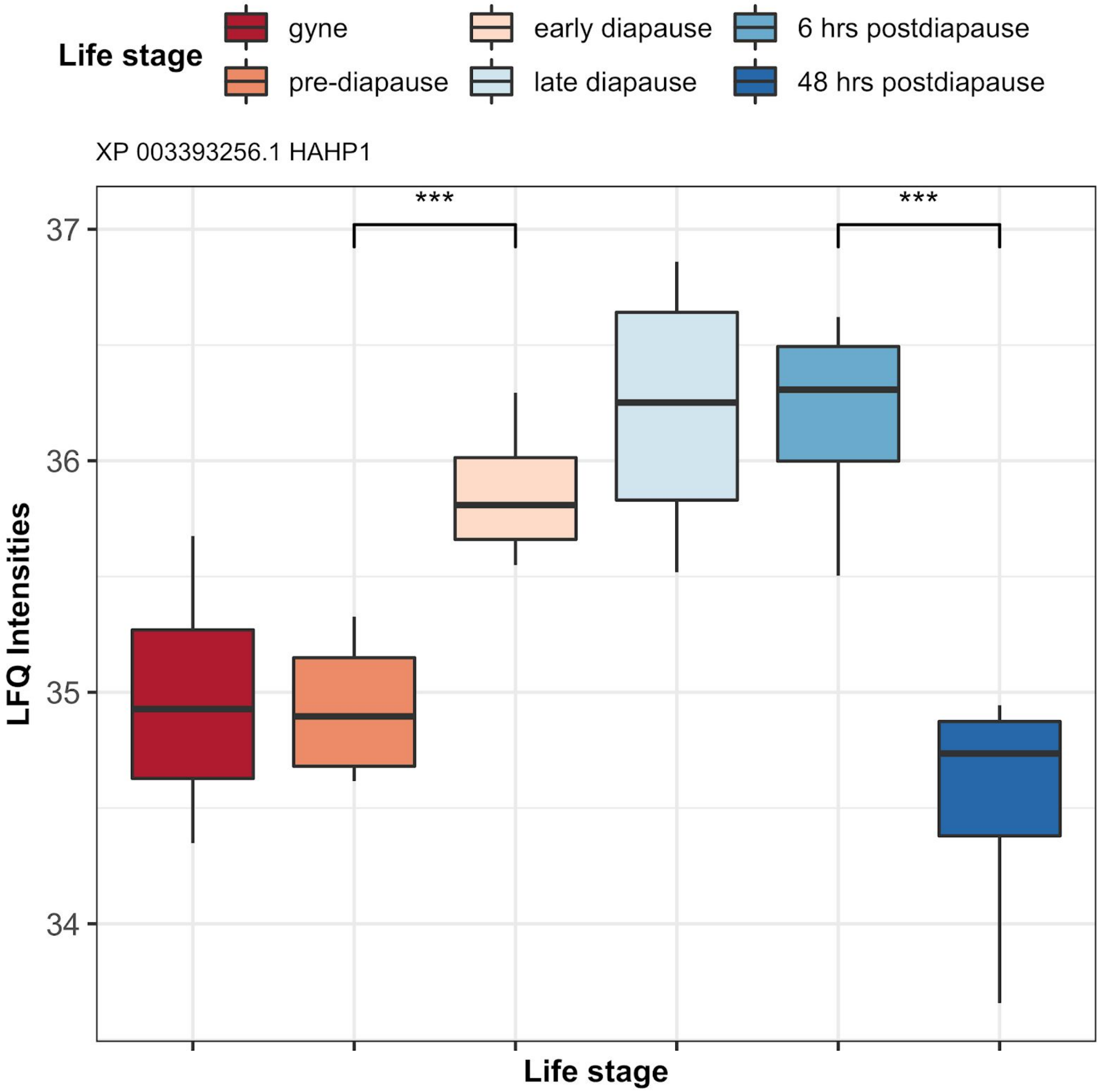

Figure S2. One HAHP family member increases in abundance during diapause. Box plots show protein abundances at six points throughout the bumblebee queen life-cycle for a novel highly abundant haemolymph-associated protein. The y-axis shows label-free quantification (LFQ) intensity values while the x-axis displays the queen life-cycle stages. Each box is coloured differently to indicate a different life-cycle stage of the queen. Stages with a significant difference in abundance (two-sample t-test; $p < 0.05$) are indicated with asterisks.

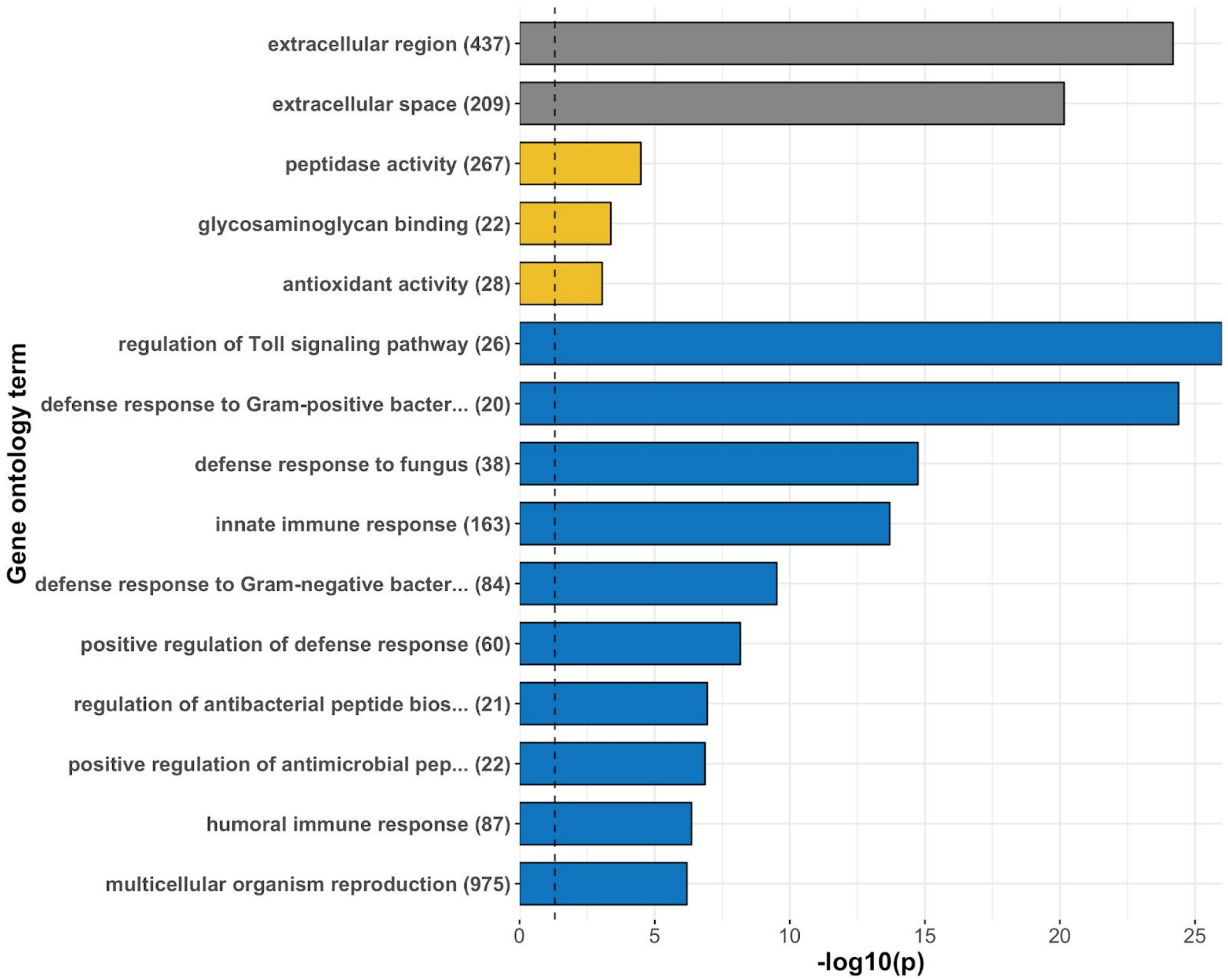

Figure S3. Gene ontology term enrichment within significantly differentially abundant proteins in the bumblebee queen haemolymph proteome. Barchart displays Gene Ontology terms significantly enriched in SSDA proteins that change in abundance throughout the queen life-cycle. For each significant GO term, the term description, as well as the total number of terms annotated within the predicted bumblebee proteome are shown on the y-axis. For each enriched term, the -log10(*p*) value is shown. Each category of GO term is displayed by an individual colour: ‘cellular component’ (grey); ‘molecular function’ (yellow); and ‘biological processes’ (blue).

References

1. Amsalem E, Galbraith DA, Cnaani J, Teal PEA, Grozinger CM. Conservation and modification of genetic and physiological toolkits underpinning diapause in bumble bee queens. Mol Ecol. 2015;24:5596–615.

2. Colgan TJ, Fletcher IK, Arce AN, Gill RJ, Rodrigues AR, Stolle E, et al. Caste- and pesticide-specific effects of neonicotinoid pesticide exposure on gene expression in bumblebees. Molecular Ecology. 2019. doi:10.1111/mec.15047.

3. Sadd BM, Barribeau SM, Bloch G, de Graaf DC, Dearden P, Elsik CG, et al. The genomes of two key bumblebee species with primitive eusocial organization. Genome Biol. 2015;16:76.

4. Bray NL, Pimentel H, Melsted P, Pachter L. Near-optimal probabilistic RNA-seq quantification. Nat Biotechnol. 2016;34:525–7.

5. Soneson C, Love MI, Robinson MD. Differential analyses for RNA-seq: transcript-level estimates improve gene-level inferences. F1000Res. 2015;4:1521.

6. Love MI, Huber W, Anders S. Moderated estimation of fold change and dispersion for RNA-seq data with DESeq2. Genome Biol. 2014;15:550.
